# Mineral composition of *Tamarindus indica* LINN (tamarind) pulp and seeds from different agro‐ecological zones of Uganda

**DOI:** 10.1002/fsn3.490

**Published:** 2017-06-29

**Authors:** Jaspher Okello, John B. L. Okullo, Gerald Eilu, Philip Nyeko, Joseph Obua

**Affiliations:** ^1^ School of Forestry Environmental and Geographical Sciences College of Agricultural and Environmental Sciences Makerere University Kampala Uganda

**Keywords:** Agro‐ecological, land use, minerals, on‐farm, *Tamarindus indica*, wild, zone

## Abstract

Mineral composition of dry *Tamarindus indica* LINN pulp and seeds was evaluated on samples collected from three different agro‐ecological zones of Uganda (Lake Victoria Crescent, and Eastern and West Nile). The objective of the study was to evaluate the mineral composition of *T. indica* pulp and seed samples from across Uganda's different agro‐ecological zones and land use types. Separately grounded samples of *T. indica* pulp and seeds were analyzed for Zn, Fe, Mg, P, Na, K, and Ca. The univariate analysis of variance in the General Linear Model was used to compare differences in mineral composition. Treatment means were separated using Least Significant Difference (LSD) in Post Hoc Tests. The results showed that there were significant differences (*p* ≤ 0.005) in mineral composition levels of *T. indica* pulp and seed samples between the different agro‐ecological zones with the exception of P and Na (for pulp). The *T. indica* pulp and seeds samples from the Lake Victoria Crescent zone and wild land use type had generally higher mineral levels than *T. indica* samples from other agro‐ecological zones and different land use types. As mineral composition levels were generally higher in the seed than the pulp samples, consumption of *T. indica* seeds should be promoted. There is also need to conserve individual species both on‐farm and in the wild population, but *T. indica* mineral concentrations (both pulp and seeds) were higher in the samples from the wild population, making them good for human and animal diets.

## 1 INTRODUCTION

Several wild tree species bear fruits and seeds during dry season. This fruiting time is very important during periods of food scarcity (Aline et al., 2008). As fruits and vegetables can provide nutrients that are needed for several human body functions (de Oliveira, des Eduardo, da Sila, & Filho, 2006), wild fruits, therefore, offer a convenient and cheap means of providing people in the tropics with adequate supplies of minerals, fat, protein, and carbohydrates. This is so because the wild and a few domesticated fruits are valuable sources of vitamins and minerals in the rural areas where exotic fruit species are limited (Amarteifio & Mosase, 2006).

Analysis of nutritional value of indigenous fruit‐bearing tree species shows that many are rich in essential vitamins and minerals while some are high in fat, protein, and crude fiber (Ochokwu, Onyia, & Ajijola, 2014). Green leafy vegetables are excellent sources of micronutrients, the consumption of these may contribute to meet the nutritional requirement and to overcome the micronutrient deficiency at minimum cost (Ebert, 2014; Saikia & Deka, 2013). Although wild fruits usually contribute to nutrition and health during the most vulnerable period of human life, data on nutritional composition of many wild fruits including *Tamarindus indica* are scanty (NRC, 2008); yet such information would be important for planning the diets of many rural communities.

Tamarind (*Tamarindus indica* LINN) and many other indigenous fruit tree species growing in various parts of the world are potentially important for supplementing diets (NRC, 2008). The *T. indica* fruits provide two important products—pulp, mostly eaten directly or used for making local food, drinks, and sold for domestic incomes, whereas the seeds, obtained after depulping the pod, are usually thrown away. According to Akajiaku, Nwosu, Onuegbu, Njoku, and Egbeneke (2014), the *T. indica* fruit pulp is acidic and has an uncommon plant acid (tartaric acid), whereas seeds are good source of protein, crude fiber, and carbohydrate. In Uganda, *T. indica* pulp is commonly used to make millet bread, porridge, juice (sweet and sour juice), and alcoholic beverages among many ethnic communities. The *T. indica* fruits are also widely sold in the local and roadside markets. Despite the above, limited knowledge on the pulp and seed nutritional values has led to low commercialization of the species (Okello, 2010).

Although studies elsewhere have documented the mineral composition of *T. indica* pulp and seeds (Akajiaku et al., 2014; Yusuf, Mofio, & Ahmed, 2007a), no attempt has been made to investigate the differences in its mineral composition between different agro‐ecological zones and land use types in Uganda. This study thus evaluated the pulp and seeds sampled from across Uganda's different agro‐ecological zones and land use types.

## 2 MATERIALS AND METHODS

### 2.1 Agro‐ecological zones of Uganda and study sites

Uganda has been zoned into 10 broad agricultural production, agro‐processing, and marketing zones for the purposes of maximizing agricultural benefits (GoU, 2004). The agro‐ecological zones are also based on agro‐climatic factors (rainfall totals and distribution) and soils (productivity and fertility). Topography, temperatures, moisture, and vegetation cover are the secondary factors considered uniform in each zone but differing between zones. In each zone, the conditions (topography, soil types, and rainfall) as well as farming systems and practices are fairly homogeneous (Mwebaze, 2002). The wet and dry seasons are fairly marked with dry season between December and March for most parts of Uganda's agro‐ecological zones. This study on *T. indica* was conducted in the three different agro‐ecological zones of Uganda where *T. indica* trees are grown both on‐farm and wild land use types. Each of the selected agro‐ecological zone was represented by the district.

Nakasongola district (Lake Victoria Crescent zone) is found in central region of Uganda covering an area of 3,510 km^2^ and altitude of between 1,000 and 1,400 masl. The district's topography is generally flat, characterized by minimal altitudinal differences with poor drainage in the wide flat valleys and shores of Lake Kyoga and endowed with unique rocky outcrops (isenbergs) (NEMA, 2004a). The vegetation type is an open deciduous savanna woodland with major geological formation of *Bululi* and *Lwampanga* soil types made of basement complex formations of the precambrian age. In this zone, there are two rainy seasons (March—July and August—November), with bimodal rainfall pattern of between 875 and 1,000 mm per annum. Most areas of the study district are very productive for agricultural and animal production, with the minimum and maximum temperatures are 18–28°C.

Soroti district in the Eastern Agro‐ecological zones is located in eastern Uganda covering an area of 3,374 km^2^. The district is found at an altitude of 1,036–1,127 masl with vegetation cover type of wooded savanna, grassland savanna, forests, and riparian. Although the major soil types are Serere and Amuria catena, Metu complex and Usuk series of agricultural productivity are moderate. Its major geological formation is granites, mignalites, gneiss, schists, and quartzites. The district is mainly underlain by rocks of the basement complex precambrian age which include granite, mignalite, gneiss, schists, and quartzite (UCC, 2003). There are two rainy seasons (March–June and August–November), with bimodal rainfall pattern between 1,000 and 1,500 mm per annum. The minimum and maximum temperatures are 18–31.3°C (GoU, 2014).

In the West Nile Agro‐ecological zone, the study was conducted in Moyo district. Moyo district is found in Northern Uganda and covers an area of 1,891 km^2^ and is at an altitude between 600 and 1,586 masl. The characteristic physical features of Moyo district include low plains as well as rolling hills and valleys that slope toward River Nile at approximately 900 masl. There is also a rise in series of hills and peaks in the northern and northeastern parts of the district (NEMA, 2004b). The major geological formation is wooded savanna vegetation type with gneiss, alluvial deposits, schist, quartzite, and marble. The schist, quartzite, and marble occur in the mountains. The major soil types are as follows: vertisols, leptosols, alluvial deposits, and ferrasols whose agricultural productivity are moderately fertile (NEMA, 2004b). In comparison to other zones, the rainfall received per annum is between 1,500 and 1,700 mm in less pronounced bimodal mainly in March–June and August–November, whereas late November to early March is a dry season. The maximum temperature (23.7–30°C) of Moyo district is higher and is of modified equatorial type (UDIH, 2007).

### 2.2 Materials

*Tamarindus indica* sampling sites were chosen from a 300 km grid as a function of the ecological gradient of the area to take into account differences in climate or ecology following Palmberg (1985). The agro‐ecological zones are located more than 300 km apart. Each agro‐ecological zone was stratified into two major land‐use types: crop fields (farmlands) and wild lands. The crop fields were current farmlands for agricultural crops, whereas the wild have not been cultivated for 5 years or more prior to the study.

The pods were collected during the fruiting season (between December 2007 and March 2008). The canopy of each sample tree was divided into three levels: top, middle, and bottom. The trees were climbed using ladders to collect ripe pods selected by gently squeezing because maturity is important in determining mineral contents, and mature ripe pods were arrived at by gently squeezing the pods. Additionally, ripe pods were considered to be those with scurfy brown, woody, fragile shell with brown pulp and blackish‐brown and hard shiny seeds (Hernadez‐Unzon & Laksminarayana, 1982).

In the three agro‐ecological zones, represented by three districts of Nakasongola, Soroti, and Moyo, four subcounties (sample sites) were selected for both land use types in a zone/district making a total of 12 study sites and covering about 5 km^2^ or more. In each sampling site, a total of five sample *T. indica* trees was selected per land use type per zone. Selection criteria included ease of access, good tree health (i.e., absence of obvious signs of pests, diseases, and fire), and presence of good mature pods. The sample trees were located at least 200 m apart, as trees close to each other are likely to have similar defects (Okello, 2010).

Eight pods were collected from each canopy level (two from each of the cardinal compass directions). In total, 24 pods were harvested from each tree following the method of Dorthe (2000). A total of 480 pods were, therefore, harvested from 20 trees per land use type, giving 960 pods per zone (district) and 2,880 pods for the two land types in all the agro‐ecological zones. Pods collected from each tree were pooled, kept in white polythene bags, and labeled according to tree number and canopy level, for example, T_1_C_1_, T_1_C_2_, and T_1_C_3_ and were taken to Makerere University's College of Agricultural and Environmental Science laboratory for analyses.

At the laboratory, the pods were washed with distilled water and allowed to dry for about 1 hr, after which manual depulping was done and morphological measurements taken. All measured samples were pooled together into land use types and agro‐ecological zones. Manual depulping of pods was carried out because the pods are indehiscence as described by Dorthe (2000). Decomposed and damaged pulp and seeds were discarded. The depulped seeds and pulp were separately sun dried for 6 hr per day for 3 days to lower the moisture content and later dried in an oven at 40°C for 3 days until the moisture content of 8% was arrived at. These were then separately grounded in an electric grinding machine (Brooks Crompton, 2000 series—UK) to 60‐mesh size based on agro‐ecological zones and land uses. The powdered samples were stored in zip lock plastic containers at room temperature for further laboratory analyses at the School of Food Technology and Bioengineering as well as the School of Agricultural Production laboratories at Makerere University. The samples were later analyzed for Zn, Fe, Mg, P, Na, K, and Ca according to procedures from Association of Official Analytical Chemists procedures (AOAC, 1999).

### 2.3 Mineral assays

Duplicate 0.5‐g pulp and seed samples were placed in digestion tubes and 7 mL of digestion mixture (composed of sulphuric acid, hydrogen peroxide, and lithium sulphate as a catalyst) added, and mixed. The mixture was heated in a block digester until the digest was clear. Heating was continued for 30 min to ensure that all the organic matter was digested, then allowed to cool to room temperature. All samples in the digestion tubes were transferred into 100‐mL volumetric flasks and made up to 100 mL using distilled water. Portions (5 mL) of the solution were then analyzed for different minerals.

The detailed analytical procedures are as described in AOAC (1999). The compositions of Zn, Fe, and Mg were determined using an atomic absorption spectrophotometer (Perkin ‐ Elmer, 2380 model) at wavelengths of 213.9, 248.3, and 285.2 nm and slit of 0.7, 0.2, and 0.7 nm for Zn, Fe, and Mg, respectively. The P composition was estimated spectrophotometrically using Jenway UV/Vis Spectrophotometer (6405 model), calibrated at a wavelength of 400 nm using blank concentrations of 0, 5, 8, and 10 ppm. Composition of Na, K, and Ca were determined using the Jenway flame photometer (model PFP 7). All mineral composition analysis was performed in triplicate and the average reading was determined.

### 2.4 Data analysis

The data were entered into Microsoft Excel by agro‐ecological zone, land use types, and source of samples (from pulp and seeds). Univariate Analyses of Variance (ANOVA) in General Linear Model was carried out in SPSS for windows version 10.0 to determine the variations in mineral composition. Treatment means were separated using the Least Significant Difference (LSD) in the Post Hoc Test. In addition, the principal component analyses were carried out.

## 3 RESULTS AND DISCUSSION

### 3.1 Mineral composition of *T. indica* pulp

The pulp mineral composition in *T. indica* was generally highest in the samples from the Lake Victoria Crescent and lowest in the samples from West Nile agro‐ecological zone. *T. indica* samples from the wild land use type had relatively higher concentration of minerals than those samples from the on‐farm land use type. There were significant differences (*p* < 0.005) in the concentration of all the minerals between the three agro‐ecological zones, with the exception of P (Table 1).

**Table 1:** *T. indica* pulp mineral composition from three different agro‐ecological zones and land use types in Uganda

Mineral Compo.‐ mg/100 g	Agro‐ecological zones and land use types												
	Agro‐ecological Zones			Land Use Types		Agro‐ecological Zones * Land Use Types						P	SE
	West Nile	LVC LVC	East LVC	Wild Land	On‐farm	LVC* Wild	LVC*On‐farm	East* Wild	East*On‐farm	WN* Wild	WN*On‐farm	zone *land	zone *land
Zn	3.3^a^	7.9^b^	2.9^a^	5.0^a^	4.4^b^	8.7	7.1	3	2.8	3.4	3.3	0.008	0.182
Fe	15.6^a^	37.9^b^	13.7^c^	20.8^a^	24.0^b^	36.8	38.9	14.7	12.8	10.9	20.4	0.001	0.578
Mg	84.7^a^	99.8^b^	95.7^c^	104.2^a^	82.5^b^	110.0	89.5	112.6	78.8	90.1	79.2	0.001	0.34
P	13.8^a^	13.5^a^	13.7^a^	13.4^a^	13.9^a^	0.3	0.2	0.1	0.2	0.3	0.1	0.001	0.502
Na	80.8^a^	171.2^b^	59.6^c^	100.8^a^	107.0^a^	171	171.3	67.7	51.5	63.6	68.0	0.001	0.019
K	20.9^a^	24.5^b^	30.5^c^	26.5^a^	24.1^b^	21.9	27.2	32.8	28.2	24.9	16.9	0.001	0.34
Ca	117.1^a^	170.5^b^	137.8^c^	155.8^a^	127.7^b^	173.6	167.3	168.3	107.2	125.5	108.6	0.001	0.004

Different letters in the same row show statistically significant differences

^*^Shows interactions.

In addition, there were also significant differences in all the mineral elements' concentration except P and Na in the different land use types (Table 1). The mineral levels of Zn, Fe, Mg, Na, and Ca were significantly higher by 31–60% in samples from the Lake Victoria Crescent zone. The P level was highest in the samples from the West Nile zone (by up to 0.7%), whereas K was more in the samples from Eastern zone by up to 20%. The composition of minerals in the samples was in the order: Ca > Na > Mg > K > Fe > P > Zn (Table 1).

### 3.2 Mineral composition of *T. indica* seeds

The composition of different minerals in *T. indica* seeds was highest in the Lake Victoria Crescent samples, while samples from the West Nile zone had the lowest values. Samples from wild had all higher values than those recorded from on‐farms samples. There were significant differences in all minerals analyzed (*p* < 0.005) between the samples from three different agro‐ecological zones. Additionally, all the mineral concentrations were significantly different in samples from the two land use types (Table 2).

**Table 2:** *T. indica* seeds mineral composition from three different agro‐ecological zones and land use types in Uganda

Agro‐ecological zones and land use types													
Mineral	Agro‐ecological Zones			Land Uses		Agro‐ecological Zones * Land Use Types						P	SE
Compos. mg/100 g	West Nile	LVC AEZ	East AEZ	Wild Land	On‐farm	LVC * Fall	LVC*On‐farm	East *Wild	East* On‐farm	WN * Wild	WN*On‐farm	zone *land	zone *land
Zn	13.2^a^	7.8^b^	6.4^c^	11.1^a^	7.2^b^	8.5	7.2	6.2	6.6	18.6	7.9	0.001	0.245
Fe	37.0^a^	30.3^b^	32.2^c^	35.2^a^	31.1^b^	33.4	27.2	33.8	30.7	38.5	35.5	0.032	0.491
Mg	196.0^a^	167.4^b^	177.6^c^	193.0^a^	167.7^b^	177.7	157	192.7	162.5	208.6	183.5	0.001	0.494
P	21.8^a^	13.7^b^	19.7^c^	22.5^a^	14.3^b^	15.6	11.8	24.9	14.4	27.1	16.6	0.001	0.116
Na	168.4^a^	109.1^b^	118.1^c^	161.3^a^	102.3^b^	116.6	101.5	153	83.2	214.5	122.3	0.001	0.005
K	11.3^a^	12.7^b^	13.9^c^	15.4^a^	9.9^b^	14.8	10.5	18.1	9.7	13.1	9.4	0.001	0.010
Ca	161.1^a^	136.1^b^	154.0^c^	170.5^a^	130.3^b^	137.2	135.1	194.2	113.7	180.1	142.1	0.001	0.005

Different letters in the same row show statistically significant differences.

^*^Shows interactions.

#### 3.2.1 Zn Concentration

Zn level was higher in the *T. indica* seeds than the pulp samples while samples from wild had higher Zn levels than those from on‐farm samples. *T. indica* samples from the West Nile agro‐ecological zone had higher Zn concentrations in the seeds than those from the other two zones. Zn levels were also higher in pulp samples from the Lake Victoria Crescent zone. Although Zn composition of *T. indica* pulp and seeds was in the ranges reported of most indigenous fruits (Valvi & Rathod, 2011), the revealed Zn levels in this study are, however, lower than those recorded in *T. indica* samples by Krithika and Radhai (2007); Chiteva and Kituyi (2006); Tariqul et al. (2013), but higher than values recorded in samples by Berto, da Silva, Visentainer, Matsushita, and de Souza (2015). As Zn exits naturally in rocks, amount present in the soil would depend on the parent materials of that soil. For example, soils originating from igneous rocks are higher in Zn (UM, 2016a).

According to Incitec Pivot (2010), Zn is present in higher amounts in clay soils, sandy and highly leached acid soils generally have low plant available Zn (not readily leached). Mineral soils with low soil organic matter also exhibit Zn deficiency (UM, 2016a). In addition, there are significant differences in the content of Zn, depending on plant species and the content in dry matter of the plants never exceed 100 mg kg^−1^ (Elżbieta et al., 2015). Plants accumulate heavy metal ions in their seeds selectively and can be taken up through leaves as the divalent ionic form (Zn^2+^) and chelated Zn. Micronutrients, such as Zn, usually limit crop growth in: highly leached acid sandy soils, organic soils, soils very high in pH, and soils that have been intensively cropped.

As an immobile element in the soil (Incitec Pivot, 2010), Zn is essential for the transformation of carbohydrates and regulating consumption of sugars. This implies that under favorable climatic and soil conditions, Zn can facilitate growth and development of the plant, promote growth hormones and starch formation, promotes seed maturation and general production, and activation of enzyme systems. The accumulation of more Zn in the seed samples in the samples from West Nile zone is probably due to the major geological formation and soil types (with more clay, sandy, and highly leached acid soils) that accumulate more Zn (NEMA, 2004b). In such a situation, accumulated Zn is taken up through leaves and stored in the seeds through Zn pumps mechanisms. Also, samples taken from the West Nile zone were from a relatively high average annual rainfall and temperature (NEMA, 2004a,b; UDIH, 2007), which could have affected the soil pH thereby increasing temperature and mineral ionization (David & Leventhal, 1995).

According to Incitec Pivot (2010), less Zn is more likely to occur on soils low in organic matter and changed wild management practices resulting into loss of soil organic matter. This is probably the reason why there is more accumulation of Zn on the samples from wild land use than on‐farm samples. In addition, the study was done during the dry season (December–March) when most tree leaves were shaded‐off. This partly explains the low Zn concentration (Zn is immobile and taken up through leaves) than those reported in studies carried out elsewhere. As the Zn composition in Uganda's *T. indica*, especially from West Nile zone samples can meet the average daily Zn requirement of 8–10 mg (Institute of Medicine, 2006), its consumption can be promoted to reduce the Zn deficiency which is reported to be common in underdeveloped and malnourished populations (Elinge et al., 2012). As Zn deficiency usually affects the immune system, healing process, taste and smell senses, and DNA and RNA synthesis (Elinge et al., 2012), promoting consumption of *T. indica* seeds or its processed products should be put at the front of planning for nutrition security in the area.

#### 3.2.2 Fe concentration

the levels of Fe was higher in the *T. indica* seeds than the pulp samples. The Fe composition (in the *T. indica* pulp and seed samples) generally exceed levels reported elsewhere (Yusuf, Mofio, & Ahmed, 2007b). While the concentration is lower than those reported by Chiteva and Kituyi (2006) and de Oliveira et al. (2006), it exceeds those reported by Ibironke, Rotimi, David, and Joseph (2006). Fe is a widely studied microelement and one of the most cited as an important nutrient for humans (Pereira et al., 2014). Fe occurs on high pH soils or certain plants and can be taken up by the leaves in Fe^2+^ and Fe^3+^ forms. An essential element, Fe operates as a component of hemoglobin, myoglobin, and cytochrome as well as a component of several enzymatic systems. This implies that Fe can play an essential role in oxygen transport and cellular respiration (Aberoumand & Deokule, 2009) as well as a catalyst in the production of chlorophyll.

As Fe is immobile, plants deficiency symptoms occur on young leaves (interveinal chlorosis), its deficiency can cause Fe deficiency anemia. Anemia usually damages muscle metabolism and may cause a decrease in learning ability and behavior problems when it occurs in children (Elinge et al., 2012). When consumed, the pulp and seeds of *T. indica* can be a good source of Fe to enrich rations for treating Fe deficiency in humans, particularly, in rural areas where *T. indica* is usually available on‐farms, in the wild population, and local markets. As the Fe values obtained in this study exceed the average daily requirement of 8–18 mg (Institute of Medicine, 2006), maintaining the *T. indica* population in the wild which have samples with relatively higher Fe levels should be encouraged to reduce pressure on the wild populations.

#### 3.2.3 K Concentration

*T. indica* K levels are higher in the pulp than seed samples, higher in Lake Victoria Crescent zone samples than in the samples from other zones and also higher in the samples from wild than on‐farm land use types. Akajiaku et al. (2014) and Ibironke et al. (2006) documented higher values of K in the *T. indica* samples than the result of this study, whereas de Oliveira et al. (2006) documented lower values of K in Brazil than those recorded in this study. As the levels of K are usually highest under osmotic stress condition (Min, Zheng, Shen, & Guo, 2013), the K supply to plant roots would depend mainly on the diffusion flux that a soil can maintain in the direction of plants to supply it. As an essential element, K, combined with sodium usually regulates the functioning of the muscle system, including heartbeats.

Averagely, *T. indica* samples from the West Nile zone had lower levels of K which is suspected as the area has high rainfall and most K could have been leached from the *T. indica* trees into the soil. In addition, K is usually used in larger amounts than any other element except N. It is not part of any structural component of the plant, located in the cell sap as an inorganic salt. In plants, K is a catalyst in many reactions, carbohydrate, starch, and N metabolism; activation of enzymes involved in photosynthesis, protein, and carbohydrate metabolism; adjustment of stomatal movement and water relations; helping in disease resistance; and increasing quality of fruits and vegetables. Because of this, it has to be obtained by plants as K^+^ from soil minerals, organic materials, and fertilizer. Due to its use in the different plant functions, K is usually less in seed than in pulp samples (Brady, 1990). Hence, consumption of *T. indica* needed to be encouraged if more K is to be taken by humans.

#### 3.2.4 P, Ca, Mg, and Na concentrations

The levels of P in *T. indica* pulp and seed samples from Uganda are generally higher than those documented in other studies elsewhere, for example, Krithika and Radhai (2007). This higher amount of P is nutritionally important because P plays important roles in many physiological processes such as membrane function, cell signaling, nucleic acid synthesis, energy metabolism muscle contractility, neurological activities, electrolyte transport, bone mineralization, normal white and red blood cell generation, and platelet function (Shaman & Kowalski, 2015). In plants, P is taken up by the plant in the form of H_2_PO_4_^−^ (primary orthophosphate) and H_2_PO_4_^2−^ (secondary orthophosphate). Consumption of *T. indica* would be highly recommended as a source of P. In plants, P functions as a part of the protein molecule; necessary for transfer of energy during metabolic processes (ATP), and hastens maturity, promotes good root development, improves drought and cold tolerance, and improves seedling vigor, important in seed and fruit formation. In addition, P is required by the plants in the formation of oils, sugars, and starches (Tariqul et al., 2013).

Ca (taken up by plants in the form of Ca^2+^) is an essential part of plant cell wall structure that provides for normal transport and retention of other elements as well as strength in the plants. In some cases, Ca, may have luxury consumption plants, may take up more than is needed. In addition, calcium pectate gives strength to cell walls, and plant root and tip elongation. In humans, Ca also plays a great role in the maintenance of bones and teeth; is involved in the transport of cellular membranes, activation or release of enzymes, and muscle contractions and transmission of nerve impulses (regulating the heartbeat) in animals.

Mg is taken up by plants in the form of Mg^2+^ and in plants, a part of the chlorophyll molecule (essential in photosynthesis), related to phosphorus metabolism and large quantities found in seed. Apart from Mg being present in high concentrations in vegetables and green parts of plants as a critical constituent of chlorophyll (Pereira et al., 2014), it is also a constituent of bones and teeth. Additionally, Mg contributes to parathyroid hormone liberation and conversion reactions of vitamin D to its active form, as well as tissue respiration, muscle relaxation, and tissue helping the growth and maintenance of bodily functions. Na may be essential for carbohydrate metabolism in some plants and may substitute for K.

The Ca, Mg, and Na values reported in this study are similar to those documented by Krithika and Radhai (2007), but higher than Ca and Mg values and lower than P and K values reported by Tariqul et al. (2013). This indicates that *T. indica* from Uganda have sufficient qualities for mineral exploitation. The relative concentrations of the different minerals recorded in this study, that is, Mg>Ca>K>P is similar to the results obtained elsewhere (Valvi & Rathod, 2011). As the levels of Ca recorded in this study are within the range documented by de Oliveira et al. (2006), the consumption of *T. indica* pulp can be promoted alone or as a cocktail juice. Generally, most minerals produced by the plants are stored in the seeds, leading to the higher mineral levels in *T. indica* seeds than the pulp samples. Stored minerals and less agricultural practices in the wild land use type probably promotes the retention of more *P*.

It should be noted that throwing away of *T. indica* seeds after depulping (a very common practice in the three agro‐ecological zones), taking away the pulp, would lead to losing a lot of nutrients as *T. indica* contain more minerals than pulp samples itself. This indicates that there are untapped potentials in the use of seeds as a source of minerals due to lack of knowledge, its availability, and postharvest handling practices. The differences in agro‐climatic factors (rainfall totals and distribution), geological formations, soils types, topography, temperatures, and vegetation cover between the agro‐ecological zones of Uganda are sufficiently great and the mineral differences are probably attributed to them. This is in agreement with Brack, Kinupp, & Sobral (2007); Kinnup & Barros (2008), who documented that the mineral content of fruits can vary according to the plant, maturity, soil conditions, climate, and agricultural practices.

Additionally, the genetic differences between *T. indica* individuals in the different agro‐ecological zones could have contributed to the observed differences in this study. As more retention of minerals in seeds was observed in the higher rainfall zone (e.g., West Nile), the levels of major minerals in fruit trees can be more in higher rainfall zones than in lower rainfall zones (UM, 2016b). Thus, the consumption of *T. indica* pulp and seed beyond the cultivation areas should be promoted to avoid Mg deficiency, which may result in neuromuscular hyperexcitation, seizures, and tetany that can also lead to death (Elinge et al., 2012). The samples analyzed in this study can serve as good natural and cheap sources of both macro and trace minerals, especially Zn, Fe, Ca, and Mg that can be promoted / used to supplement other minerals in short supply.

## 4 CONCLUSION

There were significant differences (*p* ≤ 0.005) in *T. indica* mineral composition levels recorded between the agro‐ecological zones and land use types in Uganda. The mineral levels of *T. indica* recorded in this study indicate that samples from the Lake Victoria Crescent zone and wild land use type had higher mineral levels than those recorded from other zones, land uses, and most studies elsewhere while the seed had higher mineral levels than the pulp samples. The result also shows that consumption of both the *T. indica* seeds and pulp would be highly recommended for the human and animal health as a way of overcoming macro and micronutrient deficiencies that are quite prevalent in poor urban and rural areas. Whereas *T. indica* individuals found on‐farm can be consumed, the mineral concentrations in them are lower than those from the *T. indica* in the wild population. This is attributed to the fact that there is more accumulation of organic matter in the wild land use type, which encourages accumulation of more Zn than in the on‐farm. *T. indica* seeds could therefore be promoted and developed as a good source of minerals in all the agro‐ecological zones and land use types, although its nutritional quality still need to be ascertained. While *T. indica* can be used to meet the daily requirements of minerals, especially Zn, Fe, Ca, and Mg, a comprehensive investigation of the shelf life of the *T. indica* pods which contains both the pulp and seeds needs to be done; with more emphasis on comparing *T. indica* mineral levels composition with other conventional tree pods' shelf life. This is expected to give proper guidance to the local communities interested in cultivating and consuming the species' fruit pulp. As *T. indica* samples have good mineral contents, it can also be normally included in most diet to improve human health.

## CONFLICT OF INTEREST

No conflict of interest was declared.
